# miR-129-5p targets FEZ1/SCOC/ULK1/NBR1 complex to restore neuronal function in mice with post-stroke depression

**DOI:** 10.1080/21655979.2022.2059910

**Published:** 2022-04-17

**Authors:** Fan Qinlin, Wang Bingqiao, Hu Linlin, Shi Peixia, Xie Lexing, Yang Lijun, Yang Qingwu

**Affiliations:** a Department of Neurology, Second Affiliated Hospital of Army Medical UniversityChongqing, China; b Department of General orthopedics, Chongqing Public Health Medical Treatment Center, Chongqing, China

**Keywords:** Post-stroke depression, autophagy, mice, miR-129-5p, fasciculation and elongation protein zeta-1 (FEZ1), short coiled-coil protein (SCOC), unc-51 like autophagy activating kinase 1 (ULK1), autophagy cargo receptor (NBR1)

## Abstract

Post-stroke depression (PSD) seriously affects the normal life of patients. Based on the previous sequencing results, this study selected miR-129-5p as the research object, which was significantly reduced in the PSD model by screening. To clarify the regulatory role of miR-129-5p, this study overexpressed and interfered with miR-129-5p in neuronal cells cultured in vitro, tested its effect on neuronal cell autophagy, and determined expressions of fasciculation and elongation protein zeta-1 (FEZ1), short coiled-coil protein (SCOC), unc-51 like autophagy activating kinase 1 (ULK1) and autophagy cargo receptor (NBR1) autophagy-related proteins. The dual-luciferase reporter system and immunoprecipitation were applied to detect the molecular regulatory mechanism of miR-129-5 and FEZ1, SCOC, ULK1 and NBR1. Findings of the present study revealed that the autophagy of neuronal cells was markedly decreased by overexpressing miR-129-5p (*p* < 0.05), and expressions of FEZ1, SCOC, ULK1 and NBR1 were substantially reduced (*p* < 0.05). The dual-luciferase reporter system results indicated that FEZ1, SCOC, ULK1 and NBR1 were all miR-129-5p target genes. Furthermore, immunoprecipitation assay revealed that SCOC, ULK1 and NBR1 could directly bind to the FEZ1 protein. The experiments at an animal level demonstrated that miR-129-5p could effectively alleviate the behavioral indicators of PSD model mice. Taken together, this study testified that SCOC/ULK1/NBR1 proteins could directly bind to FEZ1 to form protein complex, and all of the four proteins FEZ1/SCOC/ULK1/NBR1 were miR-129-5p target genes. miR-129-5p overexpression could effectively restore the behavioral characteristics of model mice, and reduce the autophagy-related proteins FEZ1/SCOC/ULK1/NBR1.

## GRAPHICAL ABSTRACT

**Figure:**
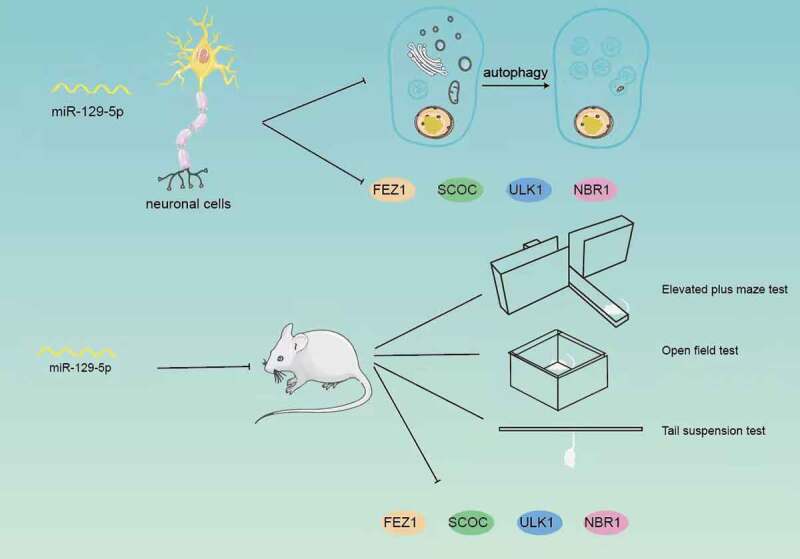

## Highlights

SCOC/ULK1/NBR1 proteins could bind to FEZ1 to form protein complex.
FEZ1/SCOC/ULK1/NBR1 were miR-129-5p target genes.
Overexpression of miR-129-5p could reduce the autophagy-related proteins FEZ1/SCOC/ULK1/NBR1.

## 1 Introduction

Stroke is currently considered to be mostly responsible for mortality and severe long-term disability worldwide. It has emerged as a serious public health problem [1]. Post-stroke depression (PSD) is the most common mental disorder after a stroke, resulting in poor quality of life and declined ability in rehabilitation of patients [2–4]. This disorder is defined as the depressive state after a stroke. A study showed that the incidence of PSD is 5–67% [5]. But the degree of depression in patients with PSD varied greatly in different studies. A META analysis of post-stroke mood disorders showed that the overall prevalence of PSD was 33.5%, with mild depression accounting for 13.10% and severe depression accounting for 17.70% [6]. And another study found that the prevalence of PSD could reach 50% within 5 years after stroke, and the incidence of mild depression and severe depression was roughly the same. Furthermore, PSD may be related to gender, and the incidence of female was higher than that of male, but the difference was less pronounced after 6 months [7]. Stroke caused physical function defects, decreased living ability, changed the family and social status, and reduced self-regulation ability of patients. Thereby increasing the incidence of PSD. Depression in stroke patients can affect their confidence and subjective initiative in participating in treatment, which not only affects the neurological rehabilitation, but also affects the recovery of cognitive function.

Current PSD research has found that acute inflammation occurs after a stroke, leading to the release of glucocorticoid [8]. These processes reduce the transcription of neurotrophic factors, thereby reducing neurogenesis and neuroplasticity, especially in the hippocampus and frontal cortex. PSD is related to cognitive dysfunction, and this disorder may be indirectly caused by hippocampal damage. The hippocampus controls cognition and emotion in a healthy brain, and hippocampal damage may be the cause of cognitive and emotional disorders. Studies have pointed out that the hippocampus contains a great many steroid receptors GRs and MRs, which mediate the control of steroids on hypothalamus–pituitary–adrenal (HPA) gland mediation of and behavior in a coordinated manner [9]. It is currently reckoned that excessive stress activates steroid receptors in the hippocampus by over-releasing steroids, thereby stimulating late neuroinflammation and signal transduction. Consequently, neurogenesis disorders and hippocampus degeneration cause depression.

The pathophysiological mechanism of PSD is unclear. Although the etiology of PSD is thought to be multifactorial both psychosocially and biologically. Multiple studies have shown that PSD may be caused by post-stroke cognitive impairment, physical disability or psychological stress caused by social isolation. More evidence has shown that multiple biological factors, such as the location of stroke, genetic susceptibility and cortex–striatum–globus pallidus–thalamic cortex projection or destruction of neurotransmitter projection pathways, and reduced neurotrophic factors or inflammation, all are associated with PSD occurrence [10–12]. As the pathophysiology of PSD is intricate, exploration of PSD pathogenesis would contribute to the development of diagnostic markers and targeted interventions in treating PSD [13–15]. In 2000, Jacobs et al. confirmed that adult hippocampal neuron damage caused depression [16]. And different studies established the animal depression models through the methods of restraint stress [17], chronic restraint stress [18] and adrenal steroids injection [19], results showed that the hippocampal neurons in all the model animals were impaired. When the hippocampal neuronal regeneration of mice was inhibited, even mild stress increased the peak level of corticosterone in plasma, which indicated that inhibition of hippocampal neuronal regeneration had a direct regulatory effect on the HPA gland (mainly regulated by the hippocampus) [20,21]. In addition, corticosteroid receptors and glucocorticoid receptors in the hippocampus, para-ventricular thalamic nucleus and anterior pituitary promoted the regeneration and differentiation of new neurons in dentate gyrus through negative feedback regulation of HPA gland [22]. Therefore, the changes of hippocampal neurons are closely related to depression.

Clinically, focal brain injury is found to be likely to induce delayed cognitive [23,24]and depressive disorders [25,26], which are recognized as comorbidity. The connection between these complications and changes in the hippocampus lacks a definite dependence upon the possibility of depression, dementia, the severity and location of the primary injury [27,28]. However, the clinical symptoms caused by this remote injury of the hippocampus cannot be ignored, and further research on the PSD pathogenesis exploration of the hippocampus is needed. Excessive corticosteroids secreted after local brain injury, especially in patients with abnormal stress response caused by hypothalamic–pituitary–adrenal axis dysfunction, interact with corticosteroid receptors in the hippocampus to induce signal pathways to stimulate advanced neuroinflammation and subsequent events, including neurogenesis and disorders of hippocampal neurodegenerative diseases. In view of this, we established a PSD model by injection of ET-1 (endothelin-1, a strong vasoconstrictor) into the medial prefrontal cortex of C57 male mice, which resulted in infarction of the medial prefrontal cortex. Sugar water was administered one week later, and elevated plus maze test, open field test, and tail suspension test were performed for behavioral experiment to verify whether the mice developed depression. The hippocampus of PSD model mice and the normal group were taken for whole transcriptome sequencing. The sequencing results revealed that miR-129-5p expression in the PSD group was greatly decreased, while the expression of FEZ1 was significantly increased. Analyzing the protein regulatory network of FEZ1, in a series of interaction proteins obtained, it is found that the three autophagy-related proteins SCOC, ULK1, and NBR1 might play a role in depression.

Some researchers have reported that miR-129-5p mediates LINC00574 and reduces the expression of UDP glucuronyl transferase (UGT2B15) in HepaRG cells through LINC00574 [29]. has-miR-129-5p has been found overexpressed in scar tissues and interacts with intraurethral fibers [30]. Meanwhile, it also links to the growth, proliferation, differentiation, adhesion, migration and apoptosis. After human epidermal stem cells are thermally injured, has-miR-129-5p can regulate wound healing. At present, there is no research on has-miR-129-5p on post-stroke depression, but our previous experiments found that post-stroke depression was significantly reduced, while FEZ1 protein was significantly increased. There is a correlation between the two. FEZ1 is the main binding partner of schizophrenia type 1 schizophrenia (DISC1), and has been involved in mediating the growth of neuronal axons and dendrites. FEZ1 gene mutations or protein structure changes may make individuals susceptible to mental disorders and cognitive disorders. It has not yet been clearly defined and has become the focus of ongoing research. Studies have found that in the injured rat model, astrocyte FEZ1 may be involved in the pathogenesis of depression after prefrontal lobe injury in rats.

From these evidences, the role of FEZ1 in mental illness and emotional cognitive impairment cannot be ignored. Despite its importance, the basic mechanism, especially its internal relationship and dynamic expression changes in post-stroke depression, is still not fully elucidated. To solve this problem, we established a post-stroke depression model by injecting endothelin (ET)-1 into the left medial prefrontal cortex of mice. We then performed behavioral assessments on these mice. Next, the FEZ1 protein of the hippocampus of this model and the changes of the three autophagy proteins that interacted with FEZ1, SCOC, ULK1, and NBR1 were evaluated by Western blot and qPCR analysis. And after regulating the changes of miR-129-5p in in vitro cell experiments, the changes of FEZ1, SCOC, ULK1, and NBR1 proteins were observed. Meanwhile, the miR-129-5p gene expression was regulated in the PSD model group, and the behavioral changes of the mice were visualized.

We hypothesized that the overexpression of miR-129-5p could restore the behavioral characteristics of model mice via reduce the autophagy-related proteins FEZ1/SCOC/ULK1/NBR1. To prove this hypothesis, HT22 cells were used as materials in vitro and behavioral testing of model mice was performed. Our results demonstrated that miR-129-5p had an important effect on neuronal cell autophagy, which its overexpression could decrease the expression of the autophagy-related proteins FEZ1/SCOC/ULK1/NBR1.

## 2 Materials and methods

### 2.1 Transfection of neuronal cells with miR-129-5p overexpression lentivirus and interference lentivirus

Neuronal cells (HT22) were obtained from American Type Culture Collection (ATCC), and cultured in 10% FBS and 1% penicillin–streptomycin in a complete cultural medium. miR-129-5p overexpression lentivirus and interference lentivirus were packaged by Chongqing Biomedicine Biotechnology Co., Ltd. 24 h prior to transfecting lentivirus, HT22 cells were inoculated into a 24-well plate at 1 × 10^5^/well. Lentifusion Max and transfection diluent were used to transfect the lentivirus into the cells. After 48 h, stable transfected strains were selected with 5 μg/mL adenine [31]. This research is approved by Laboratory Animal Welfare and Ethics Committee of Third Military Medical University.

### 2.2 qPCR detection of the expressions of miR-129-5p, FEZ1, SCOC, ULK1 and NBR1

Trizol method was adopted for total cell RNA extraction, and T5 Fast qPCR kit (Cat. # TSE301, TSINGKE) was used for qPCR test. The reaction system was: 2 × T5 Fast qPCR Mix, 10.0 μL; 10 μM Primer F, 0.8 μL; 10 μM Primer R, 0.8 μL; 50 × ROX Reference Dye II, 0.4 μL; template DNA, 0.5 μL; and ddH2O, 7.5 μL. The reaction conditions were set at 95°C, 30 s; 95°C, 5 s; 55°C, 30 s; 72°C, 30 s, 40 cycles of reaction. The primer sequences are shown in Table 1.

**Table 1.** Primer sequences

Genes	Sequences (5’-3’)
miR-129-5p-R	TCTCAACTGGTGTCGTGGAGTCGGCAATTCAGTTGAGGCAAGCCC
miR-129-5p-F	GCCAGCTTTTTGCGGTCTG
miR-qPCR-R	TGGAGTCGGCAATTCAGT
U6-F	GGTCGGGCAGGAAAGAGGGC
U6-R	GCTAATCTTCTCTGTATCGTTCC
FEZ1-F	CTTCCCTCAGAAGTTCCTGGGC
FEZ1-R	CTCAGCCTTTGTCTCAGTGCTC
SCOC-F	CAGGCGCCAGTCCTCAAG
SCOC-R	CCAGTAACTCATAGTTCAGCGGC
ULK1-F	AGCCGCATCGTCAGTCAGG
ULK1-R	AAGGCGCCGTGTCCAATC
NBR1-F	AAGGTAGTGCCCAAAGCAGTT
NBR1-R	GGTCACTGACATTGGCGGAC
GAPDH-F	AGGTCGGTGTGAACGGATTTG
GAPDH-R	TGTAGACCATGTAGTTGAGGTCA

### 2.3 The mRFP-GFP-LC3 fusion protein infected neuronal cells to trace autophagy formation

mRFP-GFP-LC3, autophagy double-labeled adenovirus, was bought from Hanbio Biotechnology Co. Ltd. 24 h before the lentivirus transfection, and the neuronal cells were subcultured in a 24-well plate pre-plated with slides at 1 × 10^5^ cells/well. The cell confluence rate of the cells on the second day was 50%~70%. The next day, the original medium was refreshed using 250 μL medium with the corresponding virus, and incubated for 2 h in an incubator. After 2 h, the medium with virus was changed using 500 μL of fresh medium. 48 h after infection, they were treated with amino acid deprivation for 2 h, followed by two cycles of washing with PBS and 4% paraformaldehyde fixation for 20 min, and photographed after loading for analysis [32].

### 2.4 Western blot detection of protein expression

The protein was lysed using RIPA lysis buffer, and BCA method was adopted to determine the protein concentration. The precast gel was removed from the refrigerator at 4°C and put into the electrophoresis tank. Subsequently, 500 μg of total protein was added to each sample and mixed by the additional of 5× SDS loading buffer at 4:1 ratio. After mixing, the protein concentration was set at 3.3 μg/μL. The metal bath was heated at 100°C for 6 min to denature the protein. The denatured total protein was taken 60 μg for loading. After running at 80 V through the concentrated glue, the voltage was adjusted to 120 V, and waited until the bromophenol blue reached the bottom of the rubber sheet without spilt out. After transferring the membrane, 5% skimmed milk blocking solution was supplied for blocking at room temperature for 1 h. Primary antibody was added for incubation overnight at 4°C, washed 3 times with TBST, 10 min each time, added secondary antibody and incubated at room temperature for 1 h, washed with TBST 3 times, 10 min each time, and exposed using ECL exposure liquid. The antibodies used were: FEZ1 (A15362, abclonal, China), 1:500; SCOC (PH309356, ORIGEN, United States), 1:500; ULK1 (A8529, abclonal, China), 1:500; NBR1 (A3949, abclonal, China), 1:500; GAPDH (A19056, abclonal, China), 1:2000; and secondary antibody (AS014, abclonal, China), 1:1000.

### 2.5 Immunofluorescence detection

Following three cycles of washing with PBS, 5 min each time, 0.5% TritonX-100 was added dropwise to the slides to punch holes at room temperature for 10 min. PBS was supplemented to immerse the Sections 3 times, 3 min each time, the slides were dropped with 10% normal goat serum, and blocked for 60 min at room temperature. After the blocking solution was absorbed with a piece of absorbent paper, diluted primary antibody at an appropriate quantity was added to each slide, placed in a humid chamber, and incubated overnight at 4°C. Subsequently, the wet chamber was removed and placed under room temperature condition for 30 min. The sections were kept in PBS for 3 min with three repeats. When the excess liquid was absorbed with a piece of absorbent paper, diluted fluorescent secondary antibodies were added dropwise, placed in the wet box for incubation at 37°C for 60 min and soaked in PBS 3 times, 3 min each time. Incubation was performed by adding DAPI drops for 5 min in the dark, the specimens were stained with nucleus, and removed excess DAPI using PBS to wash away for 5 min with four repeats. The slides were mounted with anti-fluorescence quencher and stored in a wet box [33]. Guangzhou Mingmei upright fluorescence system was applied for experimental data collection.

### 2.6 Detection experiment of dual luciferase reporter system

The vectors of dual-luciferase reporter system were constructed by Chongqing Biomedicine Biotechnology Co., Ltd. The plasmids were co-transfected into the cells, added with 50 μL culture medium free from antibiotics and serum, added 1 μg (mixed in each group) plasmid DNA, and mixed gently using a pipette; then added 1.6 μL Nanofusion transfection reagent, mixed gently using a pipette, let stand 5–20 min at room temperature, and added the cells in a 12-well plate. Distilled water was added to dilute 5*PLB (Passive Lysis Buffer) to 1*PLB, added 500 μL per well of a 12-well plate, break up the cells using a pipette, placed on a shaker at room temperature and vibrated slowly for 15 min. The lysate was aspirated into a 1.5 mL centrifuge tube for centrifugation at 4°C 12,000 rpm for 10 min, and the supernatant was transferred to a new tube. By adding 100 μL of Luciferase Assay Reagent II (LAR II) (Luciferase Assay Reagent, Cat. # E1910, Promega) working solution to the 96-well plate, 20 μL of cell lysate was supplied, mixed 2–3 times using a pipette, measured and recorded the value of Firefly luciferase, which was the luminescence value of the reporter gene. Ultimately, 100 μL Stop & Glo® Reagent (Luciferase Assay Reagent, Cat. # E1910, Promega) was added, mixed 2–3 times using a pipette, measured and recorded the Renilla luciferase value, which was the internal reference value.

### 2.7 Co-immunoprecipitation test

Pre-chilled RIPA buffer was added to the cultured cells to lyse proteins, and maintained on ice for 30 min. After being scraped from the culture dish using a pre-cooled scraper, the cell suspension was transferred to a 1.5 EP tube and centrifuged at 12,000 rpm for 10 min at 4°C. The supernatant was immediately transferred to a new centrifuge tube. Protein G/A agarose was prepared initially. Beads were washed with PBS twice. PBS was used to prepare a medium of 50% concentration. Of each 1 mL of total protein, 100 μL Protein G/A Sepharose beads (50%) was added vibrated for 10 min at 4°C to remove nonspecific contaminants and reduce background, centrifuged at 12,000 rpm 4°C for 15 min, and transferred the supernatant to a new centrifuge tube. The protein concentration was detected using BCA methods. Of 5 μL FEZ1 IP antibody was added to 500 μL of total protein. A mixture of antigen–antibody was vibrated slowly overnight at 4°C and its complex was captured following the addition of 50 μL Protein G/A agarose beads, and vibrated mildly at 4°C for 3 h, and centrifuged briefly at 2000 rpm for 5 s. The agarose beads-antigen-antibody complex was collected, followed by the removal of the supernatant, washed 3 times with pre-cooled RIPA buffer, and completely aspirated the supernatant. The complex was added with 60 μL 1× loading buffer following mild vibration [34]. The loaded sample was boiled for 5 min, free antigen, antibody, and beads were centrifuged, and electrophoresis the supernatant for Western blot detection.

### 2.8 Behavioral testing

The animal keeping and PSD model establishment were conducted as our previous study [35]. The mice in model + control virus group and model + overexpressing miR-129-5p virus group were administrated with interference lentivirus and miR-129-5p overexpression lentivirus (25 μg per mouse), respectively, by in situ injection with a stereolocator (SR-9 M-HT, Narishige). The subsequent tests were performed 7 d after injection.

Elevated plus maze test: the experimental animals were placed into the central area of the maze, with their heads toward the open arm. Each experimental animal should be placed at the same site thereafter. At the same time, the camera monitor was turned on to record the movement of the experimental animals within 5 min, and the corresponding data were recorded. During the experiment, the experimenter should be at a distance of 1 m away from the maze, and keep the experimental environment quiet free from external interference.

Open field test: the experimental animals were placed into the central area of the open field and started to record the movement of the experimental animal within 5 min, and noted the corresponding data. During the experiment, the experimenter should be at a distance of 1 meter from the open field, and keep the experimental environment quiet without external interference.

Tail suspension test: the tail of the mouse was fixed at the 1/3 part with tape and hang on a bracket. Its head was kept 15 cm away from the table, and a camera was used for photograph. The background of the camera was in obvious contrast with the fur color of the mouse. The C57 mouse used a white background and the timing was terminated after 6 min.

### 2.9 Immunohistochemical detection

The paraffin sections of the tissues at 5-μm thickness were baked overnight at 37°C, deparaffinized and incubated using 3% hydrogen peroxide for 15 min. The sections were microwaved to heat the tissue antigens, and then cooled at room temperature for 30 min. The tissue was incubated with mouse FEZ1 (1:50), SCOC (1:50), ULK1 (1:50), and NBR1 (1:100) antibodies, and all of which were obtained from Abclonal company in China. Finally, the tissue was incubated with a biotin-labeled secondary antibody (1:500, Abclonal, China) to observe protein expression.

### 2.10 Statistical analysis

All data were expressed as mean ± standard deviation. One-way analysis of variance was adopted and least significant difference test was performed for analysis. All statistical analyses were conducted via SPSS (v.18, SPSS, USA) software. The values of *P* less than 0.05 were considered the difference was statistically significant.

## 3 Results

The expression of miR-129-5p was significantly reduced in the PSD model by screening. We hypothesized that the overexpression of miR-129-5p could restore the behavioral characteristics of model mice via reducing the autophagy-related proteins FEZ1/SCOC/ULK1/NBR1. To clarify the regulatory mechanism, transfection of neuronal cells with miR-129-5p overexpression lentivirus and interference lentivirus were performed in this study. It was proved that the FEZ1/SCOC/ULK1/NBR1 were related to PSD, which changes were regulated by miR-129-5p-mediated pathway.

### 3.1 miR-129-5p reduces neuronal cell autophagy

**Figure 1.**
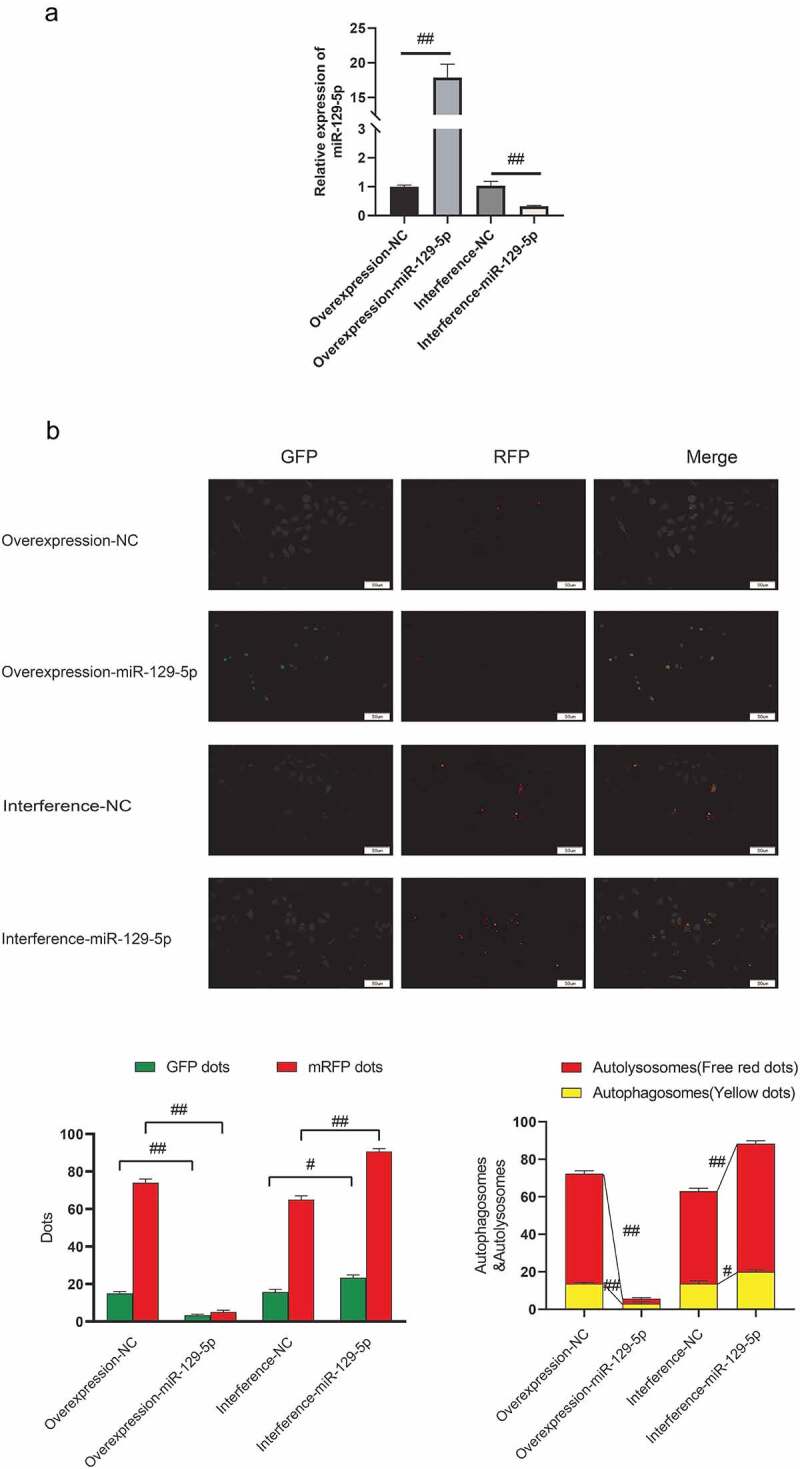
miR-129-5p reduces neuronal cell autophagy. (a) Overexpression and interference of miR-129-5p in neuronal cells cultured in vitro, and qPCR detection of miR-129-5p expression (b) mRFP-GFP-LC3 fusion protein infected neuronal cells to trace the formation of autophagy. The results followed by overexpression of miR-129-5p, the red dots of mRFP represent the degree of autophagy. Scale bar: 50 μm. #, *p* < 0.05; ##, *p* < 0.01.

In order to clarify the regulatory effect of miR-129-5p on neuronal cell autophagy, this study overexpressed and interfered with miR-129-5p in neuronal cells cultured in vitro (Figure 1a). The mRFP-GFP-LC3 fusion protein infected neuronal cells to trace the formation of autophagy. The findings suggested that after overexpression of miR-129-5p, mRFP was markedly reduced, and after interference with miR-129-5p expression, small mRFP red dots were increased markedly. The number of spots increased significantly (*p* < 0.05) (Figure 1b), indicating that miR-129-5p could minimize autophagy of neuronal cells.

### 3.2 miR-129-5p negatively regulates the expressions of FEZ1, SCOC, ULK1 and NBR1

**Figure 2.**
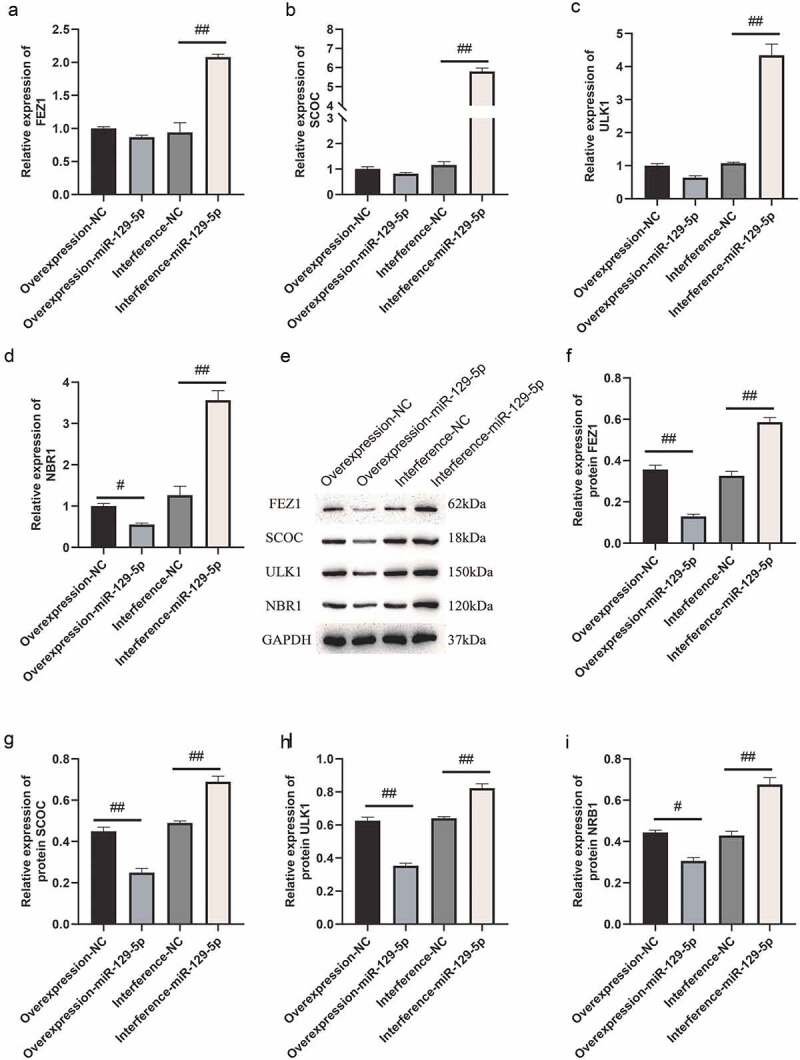
qPCR and Western blot detection of FEZ1, SCOC, ULK1 and NBR1 expressions after overexpression and interference of miR-129-5p in neuronal cells. (a) qPCR detection of FEZ1 expression. (b) qPCR detection of SCOC expression level. (c) qPCR detection of ULK1 expression. (d) qPCR detection of NBR1 expression. (e) Western blot detection of FEZ1, SCOC, ULK1 and NBR1 expressions after overexpression and interference of miR-129-5p in neuronal cells. (f) Statistical result of FEZ1 protein gray value. (g) Statistical result of the gray value of SCOC protein. (h) Statistical result of ULK1 protein gray value. (i) NBR1 protein gray value statistics results. #, *p* < 0.05; ##, *p* < 0.01.

**Figure 3.**
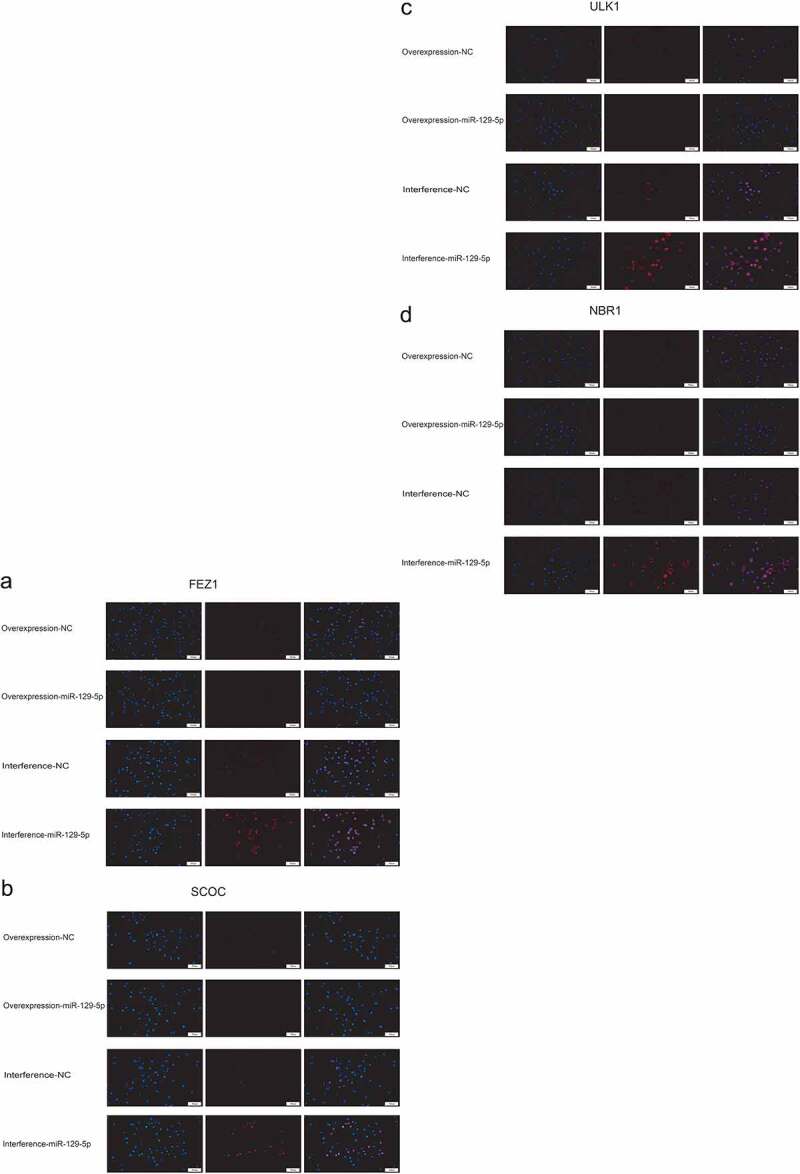
Cellular immunofluorescence detection of FEZ1, SCOC, ULK1 and NBR1 expressions after overexpression and interference of miR-129-5p in neuronal cells. (a) FEZ1 immunofluorescence results. (b) SCOC immunofluorescence result. (c) ULK1 immunofluorescence results. (d) NBR1 immunofluorescence results. (e) Statistical result of the average fluorescence intensity. Scale bar: 50 μm. #, *p* < 0.05; ##, *p* < 0.01.

Next, we used qPCR and Western blot assays to determine overexpression and interference with miR-129-5p expression, and the regulatory effects of FEZ1, SCOC, ULK1 and NBR1. qPCR assay indicated that the expressions of FEZ1, SCOC, and ULK1 tended to decrease (Figure 2(a–c)), and the expression of NBR1 was significantly reduced following overexpression of miR-129-5p (*p* < 0.05) (Figure 2d). After interfering with miR-129-5p, expressions of FEZ1, SCOC, ULK1 and NBR1 increased significantly (*p* < 0.05) (Figure 2(a–d)). Furthermore, Western blot test results revealed that all expressions of FEZ1, SCOC, ULK1, and NBR1 were significantly reduced after overexpression of miR-129-5p (*p* < 0.05) (Figure 2(e–i)). Whereas all expression levels of FEZ1, SCOC, ULK1 and NBR1 increased significantly after interfered with miR-129-5p (*p* < 0.05) (Figure 2(e–i)). Similarly, miR-129-5p was overexpressed and interfered in neuronal cells, and the expressions of FEZ1, SCOC, ULK1, and NBR1 were detected by immunofluorescence. The results were consistent with the Western blot results (Figure 3). The previously described findings indicated that miR-129-5p negatively regulated expressions of FEZ1, SCOC, ULK1 and NBR1.

### 3.3 The molecular regulation mechanism of miR-129-5p and FEZ1, SCOC, ULK1 and NBR1

**Figure 4.**
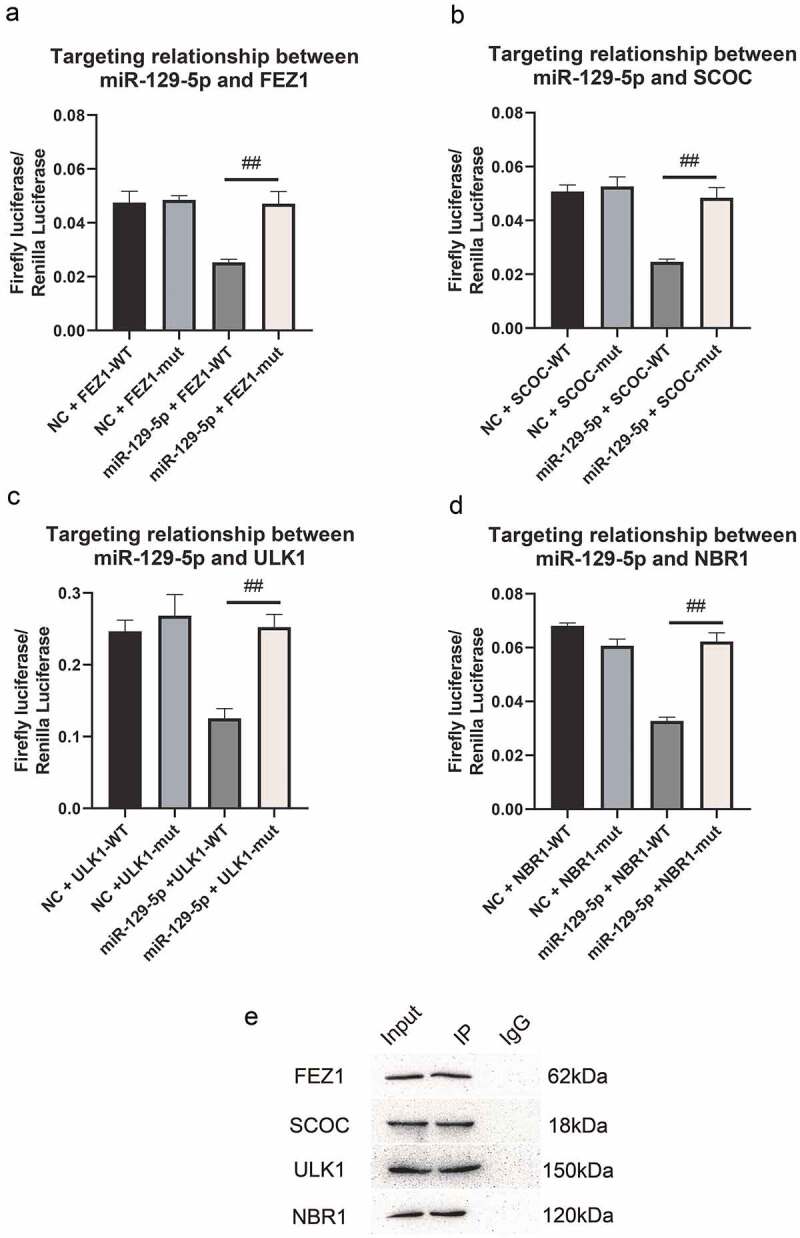
Detection of dual luciferase reporter system and co-immunoprecipitation detection. (a-d), Dual luciferase reporter system detects the targeting relationship of miR-129-5p with FEZ1, SCOC, ULK1 and NBR1, respectively. (e) Co-immunoprecipitation detection. Input is a positive control. IP is the test group, the FEZ1 protein antibody is incubated and co-precipitated, and IgG is the negative control. #, p < 0.05; ##, p < 0.01.

In our previous studies, we predicted that FEZ1, SCOC, ULK1 and NBR1 might be miR-129-5p target genes, and PPI predicted FEZ1 and SCOC, ULK1 and NBR1 interacted with each other. Therefore, in this study, we used dual-luciferase reporter system detection and immunoprecipitation test to verify their mutual regulation relationship. The results are shown in Figure 4. The dual-luciferase reporter system test revealed that after adding miR-129-5p, the expression of firefly luciferin in wild-type vectors of FEZ1, SCOC, ULK1 and NBR1 was significantly reduced (Figure 4(a–d)). It showed that miR-129-5p could bind to the 3ʹUTR region of FEZ1, SCOC, ULK1 and NBR1, and inhibited the protein translation process. It showed that FEZ1, SCOC, ULK1 and NBR1 were target genes of miR-129-5p. In addition, the results of the immunoprecipitation test showed that in the protein complexes co-precipitated with the FEZ1 antibody, the three proteins of SCOC, ULK1 and NBR1 could be detected, but these proteins were not detected in the negative control group (Figure 4e), indicating that three proteins SCOC, ULK1 and NBR1 could bind to FEZ1 protein directly.

### 3.4 Detecting the behavioral effects of miR-129-5p on depression model mice at the animal level

**Figure 5.**
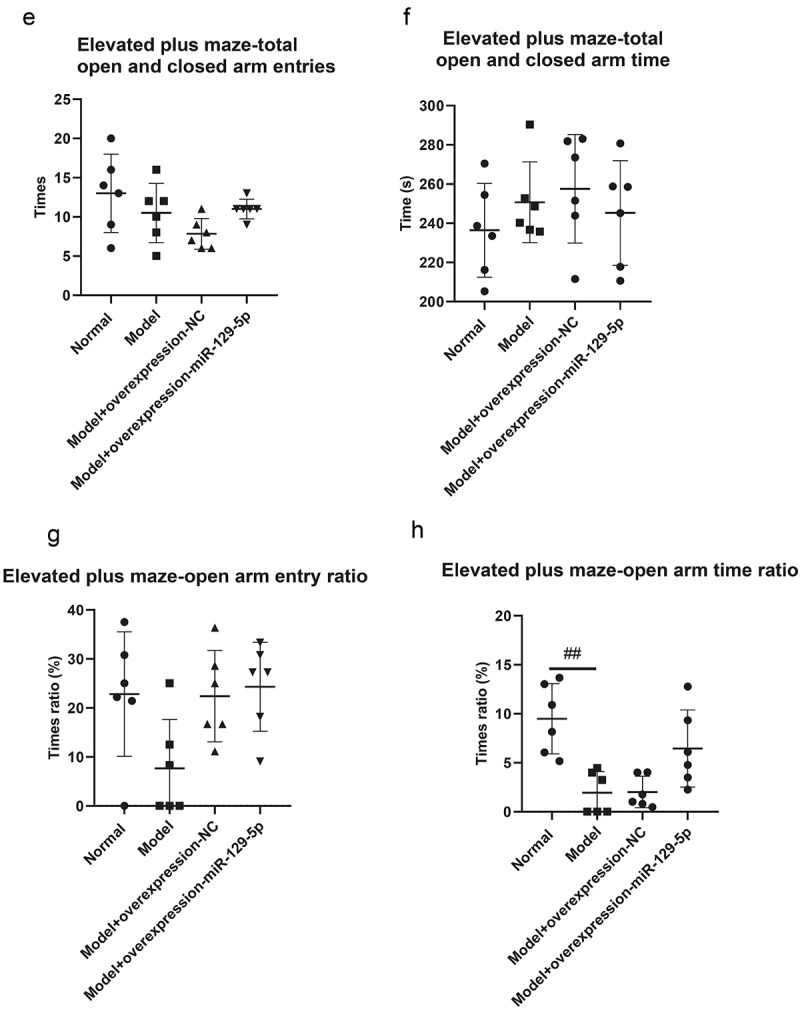
(Continued).

The elevated plus maze test revealed that the times of open arm entry of mice in normal group, model group, model + control virus group and model + overexpressing miR-129-5p virus group were 3.33 ± 2.16, 1.00 ± 1.26, 1.83 ± 1.17 and 2.67 ± 1.03 (Figure 5a); the times of closed-arm entry of mice were, respectively, 9.67 ± 3.20, 9.50 ± 3.01, 8.33 ± 1.37 and 6.00 ± 1.26 of the four groups (Figure 5b); the open arm time of mice were 21.50 ± 6.80 s, 4.83 ± 5.38 s, 4.50 ± 3.27 s and 15.33 ± 10.68 s, respectively (Figure 5c); the closed-arm time of mice were 213.33 ± 29.78 s, 244.17 ± 23.54 s, 255.50 ± 30.29 s and 226.67 ± 30.44 s, respectively (Figure 5d); the times of total open and closed-arm entries of mice in the four groups were 13.00 ± 4.98, 10.50 ± 3.78, 7.83 ± 1.94 and 11.00 ± 1.26 (Figure 5e); the total open and closed-arm time of mice were 235.83 ± 24.38 s, 251.00 ± 19.80 s, 257.50 ± 28.51 s and 244.83 ± 27.83 s (Figure 5f); Tthe open arm entry ratio of mice were 20.83 ± 11.69%, 7.33 ± 9.60%, 21.17 ± 9.45% and 23.00 ± 8.81% (Figure 5g); the time ratio of open arm entry were, respectively 9.33 ± 3.83%, 1.75 ± 1.94%, 2.25 ± 1.51% and 6.25 ± 4.17% (Figure 5h).
**Figure 5.**
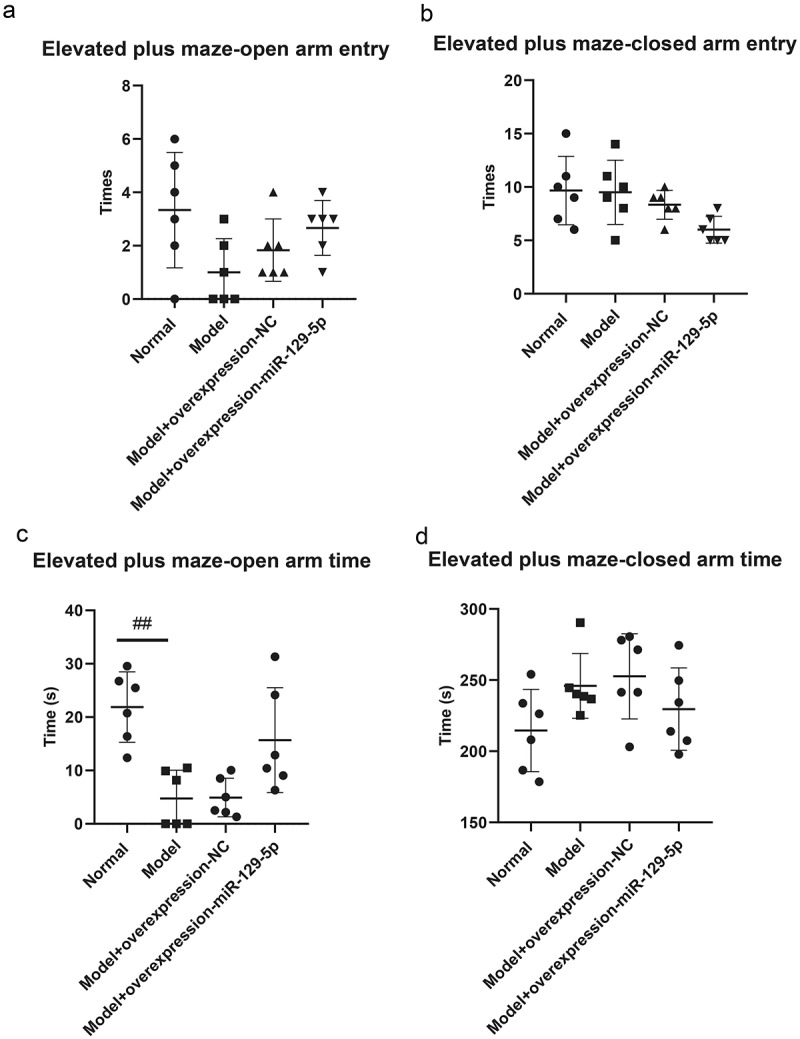
Elevated plus maze test detects the behavioral characteristics of mice in the normal group, model group, model + control virus group, and model + miR-129-5*p virus* group. ##, *p* < 0.01.

**Figure 6.**
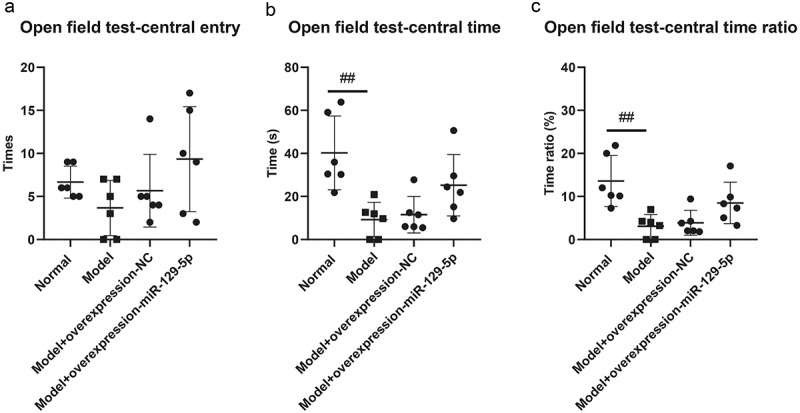
Detection of the behavioral characteristics of mice in each group via open field test. ##, *p* < 0.01.

The results of open field tests indicated that the times of entry to center in normal group, model group, model + control virus group and model + overexpressing miR-129-5p virus group were 6.66 ± 1.86, 3.67 ± 3.20, 5.67 ± 4.22 and 9.33 ± 6.09 (Figure 6a); the track time of mice in central entry were 40.00 ± 17.23 s, 9.33 ± 7.94 s, 10.17 ± 7.76 s and 26.83 ± 16.25 s, respectively (Figure 6b); the time ratio of entry to center was 13.67 ± 5.85% in normal group and 3.92 ± 3.11%, 4.42 ± 2.48%, 10.17 ± 9.06 in the rest groups (Figure 6c).

**Figure 7.**
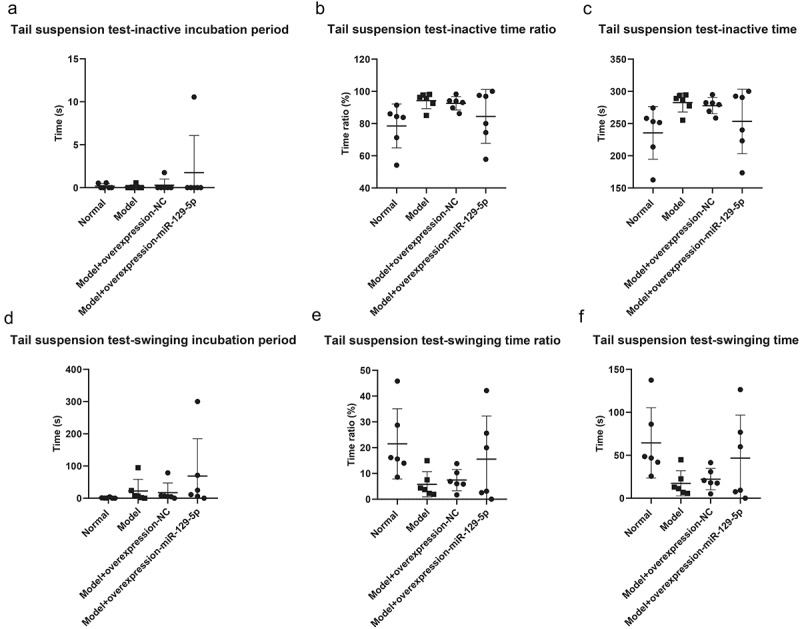
Tail suspension test to detect the behavioral characteristics of mice in the normal group, model group, model + control virus group, model + overexpressing miR-129-5*p virus* group.

The tail suspension test revealed that inactive incubation period of mice in normal group, model group, model + control virus group and model + overexpressing miR-129-5p virus group were 0.35 ± 0.54 s, 0.167 ± 0.41 s, 0.33 ± 0.82 s and 1.83 ± 4.49 s, respectively (Figure 7a); the inactive time ratio were 79.17 ± 13.96%, 92.50 ± 4.64%, 92.33 ± 4.32 and 247.67 ± 51.29 in four groups (Figure 7b); the inactive time of mice were 233.00 ± 41.46 s, 276.83 ± 12.91 s, 272.83 ± 11.02 s and 247.67 ± 51.29 s (Figure 7c); the swinging incubation period of mice were 0.83 ± 2.04 s, 24.83 ± 37.81 s, 16.17 ± 28.91 s and 65.50 ± 117.77 s (Figure 7d); the swinging time ratio of mice were 20.50 ± 13.72%, 6.00 ± 4.73%, 7.83 ± 4.02 and 15.67 ± 17.33 (Figure 7e); the swinging time of mice were 63.83 ± 39.96 s, 18.83 ± 15.75 s, 25.17 ± 13.26 s and 51.17 ± 45.27 s (Figure 7f).

Though the results of the elevated plus maze, open field, and tail suspension test did not present significant changes in the detection indicators, clearly all indicators had a recovery trend. It showed that miR-129-5p could effectively alleviate the behavioral indicators of post-stroke depression model mice.

### 3.5 Verification of miR-129-5p regulation on expressions of FEZ1, SCOC, ULK1 and NBR1 at the tissue level

**Figure 8.**
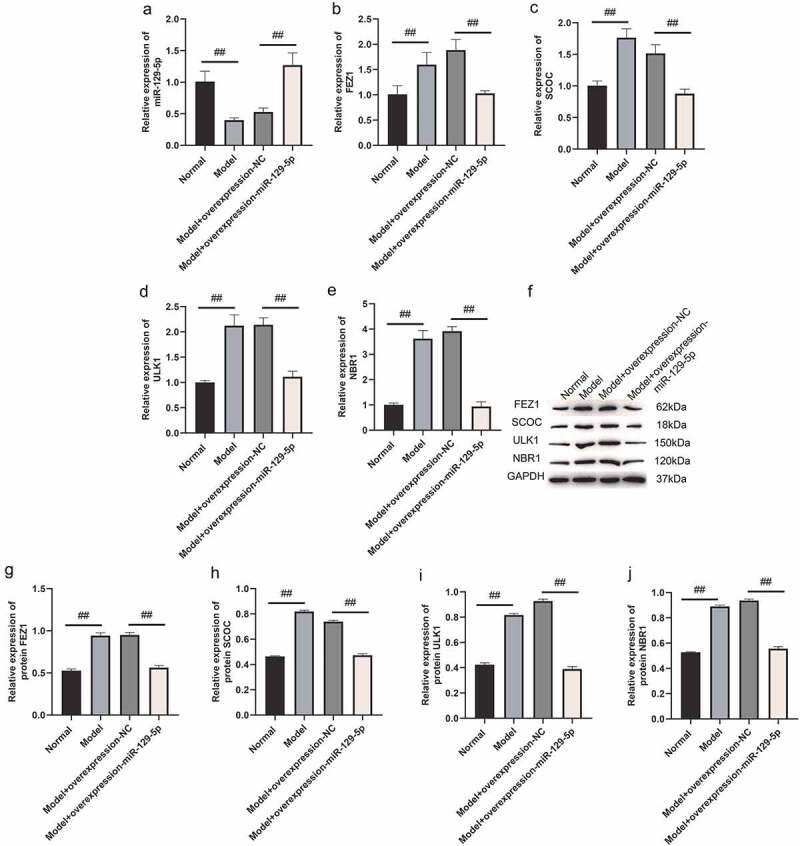
qPCR and Western blot at the tissue level verified the effects of miR-129-5p on FEZ1, SCOC, ULK1 and NBR1. (a) qPCR detection of miR-129-5p expression. (b–e), qPCR detection of mRNA expression of FEZ1, SCOC, ULK1 and NBR1. (f) Western blot detection of FEZ1, SCOC, ULK1 and NBR1 protein expressions. (g–j), Statistical result of protein gray value. ##, *p* < 0.01.

**Figure 9.**
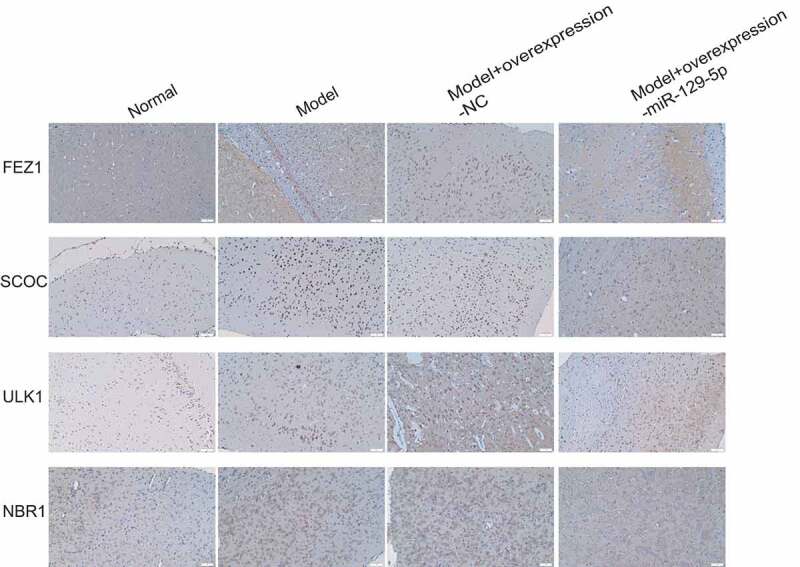
Immunohistochemical detection of the protein expressions of FEZ1, SCOC, ULK1 and NBR1 in mouse hippocampus of the normal, model, model + control virus, and model + overexpressing miR-129-5*p virus* groups.

Subsequently, we collected mouse hippocampal tissues and detected the expressions of FEZ1, SCOC, ULK1 and NBR1 after overexpression of miR-129-5p using qPCR and Western blot. The qPCR assay revealed that after overexpression of miR-129-5p (Figure 8a), the mRNA expression levels of FEZ1, SCOC, ULK1 and NBR1 were markedly downregulated (*p* < 0.05) (Figure 8(b–e)). Meanwhile, the Western blot results also indicated that after overexpression of miR-129-5p, the protein expression levels of FEZ1, SCOC, ULK1 and NBR1 were substantially decreased (*p* < 0.05) (Figure 8(f–j)). Finally, we used immunohistochemical methods to detect the protein expressions of FEZ1, SCOC, ULK1 and NBR1 in hippocampus, and the results were consistent with the trend of Western blot detection results (Figure 9).

## 4 Discussion

Post-stroke depression seriously affects the normal life of patients, and research on its pathogenesis and treatment methods is urgently needed. Based on the previous sequencing results, the present study screened miR-129-5p, which was greatly reduced in the PSD model, as the research object, and clarified its regulatory function. Consequently, overexpression of miR-129-5p could effectively restore the behavioral characteristics of model mice, and reduce the autophagy-related protein FEZ1/SCOC/ULK1/NBR1. This study found that SCOC/ULK1/NBR1 protein could directly bind to FEZ1 to form a protein complex, and all the four proteins of FEZ1/SCOC/ULK1/NBR1 were miR-129-5p target genes. miR-129-5p targeting FEZ1/SCOC/ULK1/NBR1 protein complex might provide new ideas for the research and treatment of PSD.

FEZ1 has been identified as an interaction partner of the disrupted-in schizophrenia 1 (DISC1). The latter also links to the cerebral cortex development. The growth cones of cultured hippocampal neurons contain FEZ1 and DISC1, and both can interact with F-actin. Furthermore, the synergistic effect between FEZ1 and DISC1 promotes the differentiation of PC12 cell neurons [36–38]. Therefore, FEZ1 and DISC1 molecular interaction has indicated an intimate interrelationship with neuronal pathology, FEZ1 siRNA is applied to silence FEZ1 in cultured hippocampal neurons to inhibit axon elongation [39,40] indicating that FEZ1 produces a certain impact on our neurons. In our in vitro experiments, it was also observed that the miR-129-5p and FEZ1 gene overexpression + neuronal cell group in the MTT experiment had a greater cell survival rate than the miR-129-5p and FEZ1 gene interference + neuronal cell group. Meanwhile, flow cytometry revealed that apoptosis of miR-129-5p and FEZ1 gene overexpression + neuronal cell group was markedly greater than that of miR-129-5p and FEZ1 gene interference + neuronal cell group. So our experiment also confirmed from another aspect that FEZ1 affected the survival of neurons.

Autophagy is intimately correlated with multiple individual disorders in the nervous system as it is of vital importance for the digestion and removal of residual organelles, injured proteins and cytoplasm [41,42]. Autophagy is therefore essential in maintaining the quality of proteins and organelles and supplying sufficient energy for cells. Some researchers have reported recently that depression may impair the functions of cells in autophagy [43]. Depression is related to nerve cell death. Other studies also indicate that autophagy removes injured proteins, residual organelles and cytoplasm at the onset of disease. However, when the animal is in a depression-like state, autophagy will be significantly down-regulated. ES-PE exerts a role in neuroprotection of human body. Multiple pathways were associated with depression and the effects of ES-PE, namely the AMPK pathway, which actively regulates the response of autophagy [44–46]. As an initial kinase in the autophagy pathway, ULK1 is up-regualted by AMPK to activate autophagy.

SCOC represents an effector of GTPase Arl1 of the Golgi apparatus. It has been lately recognized to play a role in positively regulating autophagy in genome-wide siRNA screening [47]. The protein is extensively and frequently expressed in expressed in the brain, heart and skeletal muscles of the human body. SCOC interacts with fascicle and FEZ1. SCOC and FEZ1 also play a unique regulatory role in autophagy [48]. The interaction of FEZ1 with the mammalian ULK1 kinase complex and relevant Drosophila homologue generates FEZ1-ULK1 complex. And when SCOC binds to FEZ1, the complex inhibits the induction of autophagy and releases autophagy. The complex SCOCFEZ1 mediates C axon growth and normal presynaptic tissue [49,50].

NBR1 is known to be a widely expressed protein with high conservation in eukaryotes. NBR1 acts as a binding partner of autophagy-related protein 8 (ATG8) family proteins in mammalian cells. p62 and NBR1 can function in selective autophagy via binding of ubiquitin and autophagosome protein [51,52]. Meanwhile, p62 links to the ubiquitin-proteasome system and the autophagy-lysosomal pathway, while NBR1 is related to the autophagy-lysosomal pathway [53,54]. As previously described, FEZ1, ULK1, NBR1, and SCOC are all related to autophagy and interact with each other. In this experiment, immunofluorescence method was conducted in the normal group, miR-129-5p and FEZ1 gene overexpression + nerve Metacell group, miR-129-5p and FEZ1 gene interference + neuron cell group and FEZ1, ULK1, NBR1, and SCOC immunofluorescence staining was carried out to these three groups, respectively. The findings indicated that following the intervention of miR-129-5p gene, the four described proteins showed similar decrease or increase, which proved once again that the four autophagy-related proteins were related to PSD and FEZ1 was the target protein of miR-129-5p gene, while ULK1, NBR1 and SCOC were FEZ1 interacting proteins.

Recent studies have bred FEZ1-deficient mice, which behaved abnormally, including excessive exercise phenotypes and increased ability to respond to psycho stimulants. Relevant abnormal mental behaviors are the result of changes in the dopamine release in the cerebral limbic pathways [55]. As the expression of FEZ1 in GABAergic neurons, its deficiency in the described neurons can change dopaminergic transmission and cause behavior abnormalities. Based on the previously described research, FEZ1 is indicated to be correlated to mental and emotional behaviors. In the present study, miR-129-5p expression was elevated in the PSD model, resulting in a decrease in FEZ1, and the depressive behavior of the PSD model mice was improved. It revealed that FEZ1 was related to the pathogenesis of PSD, which provided a basis for further exploration on the pathogenesis of FEZ1 and PSD.

Taken together, this research provides a foundation for the relationship between PSD and autophagy proteins. In recent years, increasing studies on the relationship between autophagy and depression have been carried out, but the research on the relationship between PSD and autophagy needs further and in-depth investigation to elucidate the pathway of PSD miR-129-5p gene. It can provide targets for clarifying the pathogenesis of PSD and offer reference for the treatment of PSD clinically.

## 5 Conclusion

### 5.1 Why was this study done

In our previous study, we clarified that the differential expressions of microRNAs and mRNAs in a PSD model, found that a total of 21 microRNAs expression levels were substantially upregulated, and 32 microRNAs expression levels were markedly downregulated. Thus, we drew a conclusion: miR-129-5p and autophagy-related genes FEZ1, NBR1, ULK1, and SCOC could be used as targets for further research. Thus, we performed this study.

### 5.2 What did we find and what do these findings mean

In this study, we established PSD model in C57BL/6 J mice, and used the dual luciferase reporter system and immunoprecipitation to detect the molecular regulatory mechanism of miR-129-5 and FEZ1, SCOC, ULK1 and NBR1. We found that SCOC/ULK1/NBR1 proteins could directly bind to FEZ1 to form protein complex, and all of the four proteins FEZ1/SCOC/ULK1/NBR1 were miR-129-5p target genes. These findings mean overexpression of miR-129-5p could effectively restore the behavioral characteristics of model mice and reduce the autophagy-related proteins FEZ1/SCOC/ULK1/NBR1. It might provide new ideas for the research and treatment of PSD.

### 5.3 The impact of this research on society

This research provides a foundation for the relationship between PSD and autophagy proteins. It can provide targets for clarifying the pathogenesis of PSD and offer reference for the treatment of PSD clinically.

### 5.4 Limitation of the study and the future direction

Recently more and more studies on the relationship between autophagy and depression have been carried out. Though our study provides the relations among PSD, miR-129-5p and autophagy-related protein, it is still unknown the pathway of PSD miR-129-5p gene, further and in-depth investigation needs to be elucidated. In the future, research of the mechanism of miR-129-5p gene mediated-pathway and studies of the autophagy as a potential pharmacological target for curing PSD should be carried on. There is still a lot of research to be done. Overall, this study revealed that SCOC/ULK1/NBR1 protein could directly bind to FEZ1 to form a protein complex, and the four proteins of FEZ1/SCOC/ULK1/NBR1 were all miR-129-5p target genes. Overexpression of miR-129-5p could effectively restore the behavioral characteristics of model mice, and simultaneously reduce the autophagy-related proteins FEZ1/SCOC/ULK1/NBR1.

## Data Availability

The data used to support the findings of this study are available from the corresponding author upon request.
